# Towards a coordinated approach for managing accelerated patient access to potentially beneficial medicines: reporting the perspectives of a multi-stakeholder, international workshop

**DOI:** 10.1093/haschl/qxae069

**Published:** 2024-05-22

**Authors:** Marie Phillips, Patricia Synnott, Chris Henshall, Sean Tunis, Lloyd Sansom, Daniel Ollendorf

**Affiliations:** Center for the Evaluation of Value and Risk in Health, Tufts Medical Center, Boston, MA 02111, United States; Center for the Evaluation of Value and Risk in Health, Tufts Medical Center, Boston, MA 02111, United States; Office of Health Economics, London SE1 2HB, United Kingdom; Center for the Evaluation of Value and Risk in Health, Tufts Medical Center, Boston, MA 02111, United States; Division of Clinical and Health Sciences, University of South Australia, Kent Town, South Australia 5071, Australia; Center for the Evaluation of Value and Risk in Health, Tufts Medical Center, Boston, MA 02111, United States

**Keywords:** accelerated approvals, expedited approvals, FDA, EMA, health technology assessment

## Abstract

Accelerated and conditional regulatory pathways for drug approvals are intended to enable earlier patient access to potentially life-saving treatments, or treatments that provide benefits in addressing a significant unmet need. However, there are questions about how well such pathways work, how appropriately they are applied, and how the work of regulators can be better coordinated with that of health technology assessment (HTA) and payer bodies, providers and health systems, and other stakeholders. In June 2023, a multi-stakeholder, international workshop was convened in Adelaide, Australia, to deliberate the challenges, goals, and opportunities to improve accelerated access pathways. Workshop attendees included representatives from patient organizations, regulators, HTA/payer bodies, universities (ethicists, health economists), and companies developing and marketing new medicines from Australia, Asia, Europe, and North America. We reviewed the contents of this workshop to identify areas of agreement and disagreement, report the key themes of the discussion, and delineate next steps for improving accelerated access pathways. We found that there was general agreement among workshop attendees that accelerated access could be improved significantly by strengthening processes for stakeholder coordination, and that coordinated efforts will be required to implement meaningful change moving forward.

## Background

Regulatory pathways to accelerate patient access to new treatments were originally created to address the lengthy review timelines at the US Food and Drug Administration (FDA). As a result of increased regulations for drug manufacturers, along with chronic budget and resource limitations, the FDA review timeline increased from 14 to 35 months in the early 1980s.^1^ Concerns over this time-intensive review process became more prominent during the AIDS epidemic, when potentially life-saving treatments were in development but not yet accessible to patients.^2^ Similar trends were observed in regulatory pathways worldwide. As a result, several expedited review programs were initiated by the FDA, the European Medicines Agency (EMA), and other regulatory bodies, including accelerated approval,^3^ conditional approval,^4^ and exceptional approval.^5^

There is evidence to suggest that such pathways have been successful in expediting regulatory decisions on an increasing number of potentially life-saving medicines, as well as medicines that provide benefits in addressing an unmet need. Average review times have declined significantly; by 2018, the median review time for drugs going through one of the FDA's accelerated pathways was 242 days, approximately 8 months (not including the time to full approval following the completion of confirmatory trials).^2^ The use of accelerated or conditional regulatory pathways has accordingly grown significantly; over a 10-year period ending in 2017, nearly 40% of drug approvals by the FDA or the EMA were expedited in some way.^6^ In recent years, these pathways have been used predominantly for oncology drugs, which has proven to be beneficial for certain patient populations in need of time-sensitive, life-saving care. Novel therapies for non–small cell lung cancer (NSCLC), for example, which were approved through accelerated or conditional pathways, have been associated with improved survival rates; 2-year relative survival among men with NSCLC increased from 26% among those diagnosed in 2001 to 35% among those diagnosed in 2014.^7,8^

**Figure 1.**
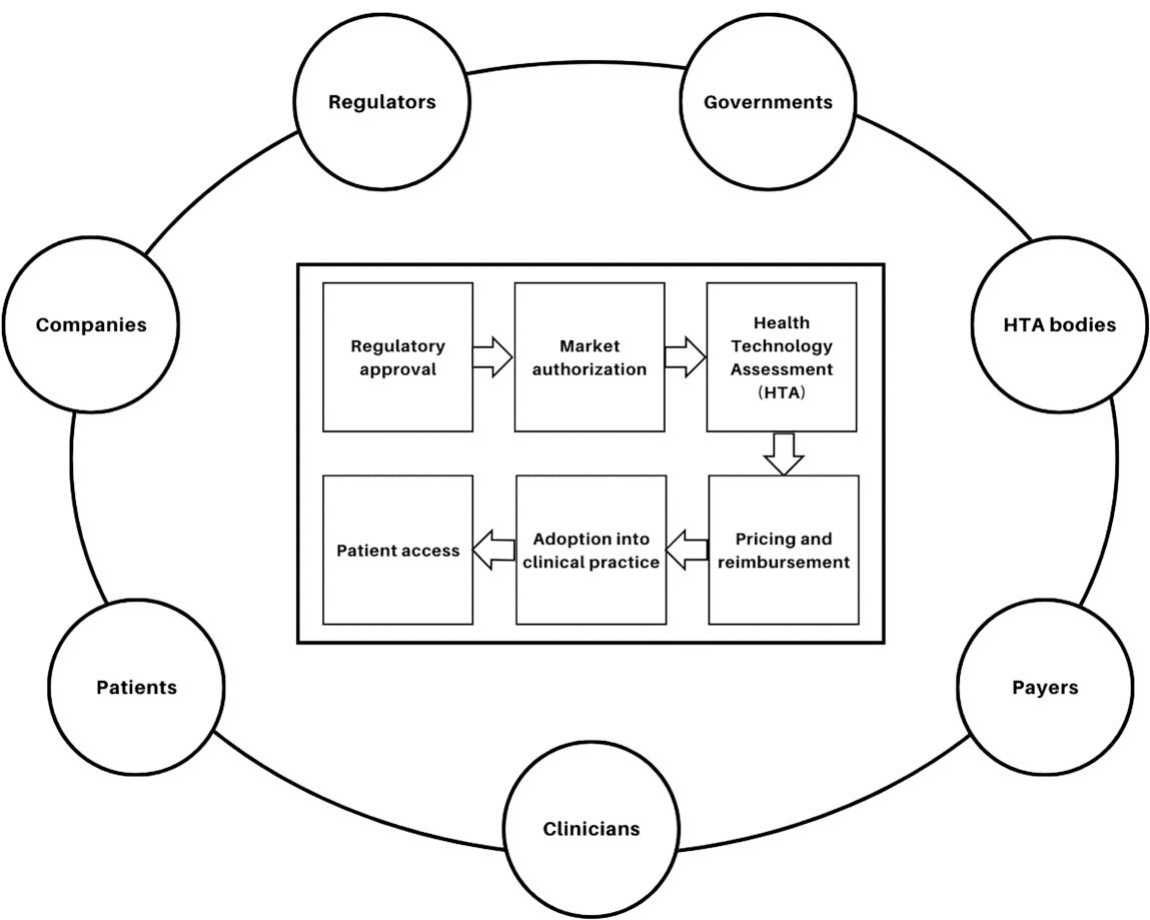
Accelerated access (AA) framework.

While earlier regulatory approval is an important first step, it alone does not guarantee earlier patient access to new treatments. Following regulatory approval, several subsequent steps must be navigated in order to facilitate access for most patients, including market authorization, health technology assessment (HTA), pricing and reimbursement, and adoption of the treatment into clinical practice. These processes involve a variety of stakeholders, including regulators, HTA bodies, payers, governments, clinicians, patients, and companies developing and marketing new medicines. These steps can be considered part of an accelerated access (AA) framework that represents a continuum from initial regulatory consideration to routine use in practice. The various processes involved in the AA framework are depicted in Figure 1.

There are questions over how well AA pathways work overall, and how the challenges can be addressed. First, the limited level of evidence initially available for AA drugs, which often comes from small, short-term, and/or uncontrolled studies, presents challenges for HTA agencies, payers, and health systems in determining effectiveness relative to available alternatives and value. This may, in part, explain why in the 10-year study of FDA/EMA accelerated or conditional approvals described above, fewer than half of the drugs were rated as having high therapeutic value.^3^ Additionally, drugs are granted accelerated or conditional regulatory approval on the premise that longer-term studies to confirm clinical benefit will be completed, yet a number of such studies have exceeded their proposed timelines and benefit remains unconfirmed.^9^

In this paper, we report the findings of a multi-stakeholder international workshop convened in Adelaide, Australia, from June 20 through June 23, 2023, to deliberate on the benefits, challenges, goals, and principles of AA, and to identify opportunities for improvement.

## Meeting structure

The multi-stakeholder workshop was organized by Bellberry, Ltd, a not-for-profit organization that promotes the welfare of research participants and the quality, efficiency, and effectiveness of research.^10^ Meeting attendees included 58 representatives from patient organizations, regulators, HTA/payer bodies, universities (ethicists, health economists), and companies developing and marketing new medicines from Australia, Asia, Europe, and North America (see Table 1 for names and affiliations).

**Table 1.** List of workshop attendees.

	Title	Organization
Dr Nitin Bagul	Principal Medical Advisor, Prescription Medicines Authorisation Branch	Therapeutic Goods Administration (TGA)
Ginny Beakes-Read	Executive Director, Global Regulatory and R&D Policy	Amgen
Meindert Boysen^a^	Head of International Affairs	National Institute for Health & Care Excellence (NICE)
Andrew Bruce^a^	Director, Global Health Policy	Amgen
Dr Eddie Cliff	Post Doctoral Fellow	Harvard Medical School
Dr Dávid Dankó^a^	Partner	Ideas & Solutions
Elizabeth de Somer	Chief Executive Officer	Medicines Australia
Dr Lisa Eckstein	Director of Philanthropic Activities	Bellberry Limited
Prof Hans-Georg Eichler^a^	Consulting Physician	Association of Austrian Social Insurances
Dr Yasu Fujiwara	Chief Executive	The Pharmaceuticals and Medical Devices Agency (PMDA)
Dr Ramiro Gilardino	Global HTA & Access Policy Leader	Merck Sharp & Dohme (MSD)
Christoph Glaetzer	Chief Global Value & Access Officer	Johnson & Johnson
Prof Wim Goettsch	Special Advisor HTA	National Health Care Institute, The Netherlands
Adrian Griffin^a^	Vice President, HTA & Market Access Policy	Johnson & Johnson
Beth Hamilton^a^	Vice President, Global Oncology Access, Pricing & Health Economics	AstraZeneca
Dr Chris Henshall^a^	Workshop Facilitator	
Prof Kirsten Howard	Co-Director, Menzies Centre for Health Policy and Economics	The University of Sydney and Pharmaceutical Benefits Advisory Committee (PBAC)
Dr Hong Ju	Senior Principal Lead Specialist/Deputy Director	Agency for Care Effectiveness (ACE)
Anna Kaltenboeck	Principal	ATI Advisory
Prof Ian Kerridge	Professor of Bioethics and Medicine	The University of Sydney
My-Linh Kha	Senior Vice President & General Manager, Japan Asia-Pacific (JAPAC)	Amgen
Koshin Kiyohara	Office Director, Office of Review Management	The Pharmaceuticals and Medical Devices Agency (PMDA)
Esther Krofah	Executive Vice President, Health	Milken Institute
Prof Wendy Lipworth	Professor of Bioethics	Macquarie University
Prof Tracy Merlin	Director	Adelaide Health Technology Assessment (AHTA)
Dr Nicole Mittmann^a^	Chief Scientist & Vice President of Scientific Evidence, Methodologies and Resources	Canadian Agency for Drugs and Technologies in Health (CADTH)
Dr Michelle Mujoomdar^a^	Manager, Policy, Non-Insured Health Benefits Program	Indigenous Services Canada
A/Prof Kwong Ng	Chief HTA Officer	Ministry of Health
Ian Noble	Director, Value Access & Policy, Australia	Amgen
Dr Liza O’Dowd	VP, Global Policy & Regulatory Intelligence, and Global Regulatory Affairs, Immunology	Johnson & Johnson
Dr Dan Ollendorf^a^	Workshop Scientific Secretariat	Tufts Medical Center
Brian O’Rourke	President	BOHCC
Marie Phillips^a^	Workshop Scientific Secretariat	Tufts Medical Center
Dr Franz Pichler	Principal & Founder	Confluence Health Consulting
Dr Francesco Pignatti^a^	Scientific Adviser—Oncology	European Medicines Agency (EMA)
Prof Andrew Roberts AM	Metcalf Chair of Leukaemia Research	University of Melbourne
Alexander Roediger^a^	Associate Vice President, Global Oncology Policy	Merck Sharpe & Dohme (MSD)
Dr Murray Ross	Senior Instructor	Kaiser Permanente School of Medicine
Prof Libby Roughead	Research Professor	University of South Australia
James Ryan	Group Director, Health Economics and Payer Policy	AstraZeneca
Dr Bettina Ryll^a^	Founder	Melanoma Patient Network Europe
Prof Emeritus Lloyd Sansom AO^b^	Director	Bellberry Limited
Prof Ingrid Scheffer AO^a^	Director	Bellberry Limited
Dr Joshua Sharfstein	Professor of the Practice in Health Policy & Management	John Hopkins Bloomberg School of Public Health
Ann Single	Coordinator	Patient Voice Initiative
Kylie Sproston^a^	Chief Executive Officer	Bellberry Limited
Patricia Synnott^a^	Workshop Scientific Secretariat	Tufts Medical Center
Prof Adrian Towse^a^	Emeritus Director and Senior Research Fellow	Office of Health Economics
Dr Sean Tunis^a^	Workshop Scientific Secretariat	Tufts Medical Center
Julie van Bavel	Executive Director, Centre for Observational and Real World Evidence, Asia Pacific	Merck Sharp & Dohme (MSD)
Dr Tina Wang	Senior Manager—HTA Programme and Strategic Partnership	Centre for Innovation in Regulatory Science (CIRS)
Jo Watson	Deputy Chair	Pharmaceutical Benefits Advisory Committee (PBAC)
Stefan Weber	Global Head of Policy	AstraZeneca
Leanne Weekes	Director of Research, Policy & Advocacy	Bellberry Limited
Prof Andrew Wilson^a^	Chair	Pharmaceutical Benefits Advisory Committee (PBAC)

Abbreviations: HTA, health technology assessment; Prof, Professor.

^a^Scientific Advisory Board member.

^b^Scientific Advisory Board Chair.

The workshop's Scientific Secretariat included academic researchers from the Center for the Evaluation of Value and Risk in Health at Tufts Medical Center (Boston, MA, USA). The Secretariat prepared a background paper on accelerated and conditional regulatory pathways, co-facilitated the workshop, and developed a meeting report summarizing the issues raised at the meeting and next steps for improvement. A Scientific Advisory Board recruited by Bellberry met monthly in the year leading up to the workshop to develop the meeting agenda and invitation list, and to review the background paper. Meeting participants agreed to use the term “accelerated access (AA) framework” to refer to the various processes involved in enabling earlier patient access to new drugs. These steps, as depicted in Figure 1, include regulatory approval, market authorization, HTA, pricing and reimbursement, and adoption into clinical practice. The AA framework referred to in this paper encompasses these various processes along with the broader ecosystem of stakeholders involved in them, including regulators, governments, HTA bodies, payers, clinicians, patients, as well as companies developing and marketing new medicines.

The workshop began with an overview of the literature on expedited regulatory pathways for drug approvals. Stakeholder representatives presented on the benefits and challenges of AA from each of their perspectives, including patient, regulatory, HTA/payer, companies developing and marketing new medicines, and ethical/societal views. On day 2, a drafted statement of goals and principles for AA prepared by the workshop Secretariat and Scientific Advisory Board was presented, and meeting delegates discussed their points of agreement and disagreement in breakout groups. Delegates then shared their feedback on the goals and principles during a plenary discussion, as well as what responsibilities each stakeholder has in order to develop a more coordinated approach to AA. The Secretariat presented a summary of this feedback on day 3, followed by a discussion on future action items to raise awareness of the issues discussed. On the final day of the workshop, members of the public were invited to receive a summary of the workshop discussion, share their own perspectives, and provide feedback for the meeting outputs. Participants agreed to follow the Chatham House Rule, which states that “participants are free to use the information received, but neither the identity nor the affiliation of the speaker(s), nor that of any other participant, may be revealed.”^11^

Importantly, this paper captures key points discussed during the workshop, but should not be construed to be a consensus statement from workshop attendees.

## Meeting proceedings

### Stakeholder perspectives

Throughout the workshop, there were multiple presentations and discussions regarding the challenges and benefits of AA for each stakeholder group involved. While participants acknowledged that the ideal practices surrounding AA may vary among different societies, there was wide agreement that a more coordinated approach to AA requires all relevant perspectives to be considered. The stakeholder perspectives voiced at the workshop are summarized in the sections that follow.

#### Patients

Patient representatives at the workshop emphasized that earlier access to novel therapies can be life-saving. While they acknowledged that risk preferences vary between individuals, representatives expressed that patients are generally willing to accept greater uncertainty about the balance of safety and effectiveness when there is a significant unmet need. Specifically, patients experiencing severe and life-threatening illnesses are generally inclined to accept a more uncertain safety profile. However, patient representatives expressed that they are opposed to accepting lower standards of evidence for regulatory and reimbursement decisions. In addition, patient representatives were opposed to policymakers creating pre- and postmarket evidence requirements that unnecessarily delay access and therefore compromise the patient.

Patient representatives also emphasized the need to develop flexible regulatory and payment systems to allow for quicker adoption of novel therapies. One example discussed was that of checkpoint inhibitors, which diverge from conventional cytotoxic chemotherapies by leveraging the immune system to fight cancer. The novelty of this treatment indicates a certain extent of uncertainty regarding the risk–benefit profile; however, checkpoint inhibitors have provided sustained clinical response for some patients, who may not have had access to these treatments if the uncertainty had caused delays in the regulatory and payment processes. In addition, patient representatives urged regulators and payers not to restrict AA programs to first-in-class medicines, since later-generation drugs in the same class may also provide important improvements in safety and efficacy.

In addition to improved flexibility surrounding regulatory and payment systems, patients noted the need for decision-makers to improve their communications regarding the benefits and risks of drugs following an AA pathway, as well as key uncertainties in the evidence. Finally, patient advocates voiced the importance of prioritizing affordability for individual patients and the health system as a whole.

#### Companies developing and marketing new medicines

Representatives from companies that develop and market new medicines emphasized the importance of innovation in driving improved outcomes and positive changes in the standard of care. They voiced the need to bring innovation to patients in a timely and efficient manner without reducing standards for demonstrating efficacy and safety. A key challenge in bringing innovation to patients rapidly is the variability in health system dynamics around the world; standards of care differ across jurisdictions, which presents competing and divergent evidence demands from regulators, HTA bodies, and payers from different countries. There is also variability in the preferences and priorities of different societies, the extent to which jurisdictions are willing to pay for health improvements, and reimbursement policies. These differences pose a challenge for enabling earlier patient access to new therapies.

Company representatives noted that regulatory agencies have taken steps to coordinate reviews and evidence requirements across multiple jurisdictions; however, they would like to have more coordination between HTA organizations. Health technology assessment organizations scope their evaluations differently, and companies developing new medicines are challenged to align their clinical development plans with these variations in HTA frameworks. Inconsistent evidence requirements contribute to delays in patient access, and AA could improve by defining commonalities in data needs. Company representatives also cautioned that too much effort and resources focused on coordination could have the unintended consequence of delaying access as well. Last, company representatives appreciated regulators' adaption to fast-moving science by accommodating novel endpoints, synthetic controls, and real-world evidence, and stressed the need for HTA organizations and payer processes to similarly adapt to new science and methodology.

#### HTA and payer organizations

The HTA and payer organizations acknowledged that their review processes are resource intensive and may contribute to delays in access. Health technology assessment organizations are responsible for making judgments about the therapeutic value and/or long-term value for money of a health technology. These judgments require reasonable certainty that a therapy provides meaningful clinical benefit. However, evidence to support such judgments is limited for AA therapies at the time coverage determinations are made. To complicate matters, the design of confirmatory trials often does not reflect the needs of HTA organizations and payers, limiting their ability to update their recommendations when the results become available. Some HTA organizations have experimented with collaborating with regulators to provide joint scientific advice to companies that develop and market new medicines. They noted that additional coordination could help them more efficiently address uncertainties.

Affordability was another important theme raised by HTA and payer representatives, who are concerned about paying high prices for medications that may ultimately turn out to be ineffective or unsafe. Representatives from HTA organizations noted that AA therapies present greater financial risk for a payer, which can mean greater health opportunity costs for the populations they serve. There was interest in adopting mechanisms for sharing financial risk and managing other elements of uncertainty, such as with studies of the long-term real-world effectiveness of the drug. A few representatives suggested that managed entry agreements could provide a framework for risk-sharing arrangements that address clinical and financial uncertainty, although the variable success of such programs was noted.

#### Regulatory agencies

Regulatory representatives at the workshop, including participants with current or previous experience with the EMA or FDA, emphasized that AA is not intended to subvert basic standards of evidence or provide the “right to try,” for which separate pathways (eg, investigational use) exist. Like their HTA counterparts, regulators encounter tradeoffs between facilitating faster access to medicines and ensuring there is reasonable certainty about a positive risk–benefit profile for those products. There was general agreement that early access should not compromise the ability to definitively know whether a drug safely provides a benefit to patients.

An additional challenge for regulators is incentivizing the timely completion of confirmatory studies. While the FDA and other agencies have established mechanisms to encourage compliance from drug companies, delays in the completion of these definitive studies sometimes occur. Regulators pointed out that delays are not always related to a lack of compliance from the drug company; rather, post-approval enrollment in clinical trials presents unique ethical and feasibility challenges for patients who may not wish to risk being randomized to an inert or potentially inferior therapy.

### Goals and principles of a functional AA system

The workshop Scientific Secretariat created a draft set of goals and principles for improving AA prior to the workshop. During the workshop, the goals and principles were presented, deliberated in breakout groups and plenary sessions, and redrafted. The original goals of AA in the draft statement were as follows: “to allow patients faster access to new prescription medicines that appear likely to offer significant benefits for life threatening or severe conditions with no alternative treatments, and to improve incentives for pharmaceutical companies to develop such medicines, whilst protecting patients, health systems, and society from unintended harm.” These were supported by 5 fundamental principles that focused attention on the legitimate, but differing, objectives that each stakeholder in AA has, inclusion of all stakeholders in the design and implementation of AA processes, and the desire to make AA complementary to rather than a replacement for traditional access pathways, and 9 operational principles that described the coordination necessary across all AA stakeholders as well as specific duties and responsibilities for each of them.

Some points of agreement among workshop attendees emerged in discussion surrounding the draft goals and principles. There was widespread agreement that the AA ecosystem could be improved through more effective “handovers” between stakeholders (eg, between regulators and HTA bodies), and increased communication and data-sharing. Also, there was agreement that patients and patient advocates should be involved earlier in the AA process, and should be integral to decisions about clinical development, outcome measurement, and timing of confirmatory trials. Finally, attendees agreed that discussion surrounding evidence generation should focus on approaches that are plausible, reasonable, economically feasible, and necessary.

There were differences of opinion regarding the overarching goals of the discussion. Some participants felt that the regulatory component of AA had been solved and the focus should be on how the rest of the system should adapt. Others felt that the construct of some accelerated or conditional regulatory pathways introduced too much risk of misuse (eg, as a rescue platform for failed clinical development) or provided too strong of an incentive for developers of medicines to apply for these pathways in situations where a traditional approach featuring randomized controlled trials is both feasible and ethically appropriate. There was a larger discussion of these incentives, as some felt that a too heavily incentivized system could cause undue harm to “traditional” (ie, not accelerated) access pathways for other medicines and health needs.

Discussions of guardrails that could be added to the system also produced different views. Some felt that real-world data should be used to not only reduce clinical uncertainty but also to adjust pricing, while others felt that these methods are not yet rigorous enough for that purpose. Some participants also felt that a clear timeline should be communicated for reduction in uncertainty that would satisfy patients, clinicians, and payers, but others felt that such uncertainty was a system feature, which should be tolerated as long as promised confirmatory trial timelines were adhered to. Discussion of surrogate endpoints followed a similar path; some participants felt that there should be more clarity and justification for why a particular surrogate was chosen, whereas others felt that AA pathways by definition are triggered in a setting where relatively little is known and the surrogates that are chosen represent the best current understanding of a connection to patient-centric outcomes.

Other points raised were that any meeting statement could draw attention to common misunderstandings about AA—for example, that AA pathways undermine safety and risk assessments in order to fast-track new drugs to patients. It was also suggested that any statement should refer to scheduling early, collaborative conversations with stakeholders as a means to improve system efficiency. Finally, there were suggestions that any meeting statement should refer to the need to avoid inequities in patient access that may arise as a result of the way that AA operates in practice, both in terms of access to medicines approved through AA pathways (which may lead some payers to restrict or withhold coverage) and more generally through pressures that AA may place on payers to give priority to funding medicines of unproven effectiveness.

### Opportunities for improvement

Following discussion of the challenges that exist surrounding AA as well as the goals and principles of a well-coordinated AA system, workshop attendees suggested specific steps that could be taken by each relevant stakeholder to optimize an AA system. Some attendees suggested that governments should play a role in establishing the overall goals of AA, coordinating stakeholder activity, and brokering communication. Attendees appeared to agree that regulators can take steps to facilitate more communication with other stakeholders and provide more clarity and consistency in the intended use of AA pathways. There was discussion of how HTA bodies and payers can increase activity around evidence generation and risk sharing, including alternative payment mechanisms (eg, pay-for-performance, milestone, clawback, etc). Most of the suggestions for companies developing and marketing new medicines focused on clear and public communications. With regard to patients and clinicians, most of the discussion involved ways they can be incorporated into measurement, methods, and communication around AA technologies. Table 2 lists the opportunities discussed for each stakeholder to contribute to an improved AA system, including priority actions and longer-term modifications.

**Table 2.** Opportunities for improvement: priority actions and longer-term modifications.

**Government**
Priority actions Ensure coordination of all players in the AA pathway; provide incentives for appropriate engagement Review policies or regulations that inhibit information-sharing between AA participants Ensure resources are available for the collection of post-licensing real-world or other data without undue burdens on clinicians and health care facilities Longer-term modifications Set the overall policy paradigm for AA for all key stakeholders in the AA pathway Ensure that AA policy supports both rapid entry and rapid exit of technologies on the AA pathway Develop specific approaches to integrate health equity considerations in AA pathway and recommendations
**Regulators**
Priority actions Develop guidance on appropriate surrogate outcome measures at the disease level as well as methodologic approaches where there are evidence generation challenges Provide parallel or joint scientific advice with HTA bodies and payers whenever possible After initiation of an expedited pathway, but prior to approval, participate whenever possible in meetings with HTA/payer organizations and health technology developers to discuss uncertainties in the available data, the plausible links between surrogate and patient-centric outcomes, and plans for post-licensing evidence generation Build on recent efforts to ensure confirmatory studies are underway at the time of accelerated or conditional approval, provide regular updates on the progress of these studies, and set parameters for enforcement if milestones are not met Provide public explanations of the scientific analyses that were conducted as well as justification for the decisions made Consider standard interactions with stakeholder community Longer-term modifications Provide clear explanations about what AA pathways are for, and what they are not for Provide clear and understandable criteria for the types of medicines eligible for AA
**HTA bodies/payers**
Priority actions Develop explicit approaches to integrate health equity considerations in the AA policy paradigm and recommendations Review policies or regulations that inhibit information-sharing between AA participants Ensure resources are available for the collection of post-licensing real-world or other data without undue burdens on clinicians and health care facilities Longer-term modifications Develop an explicit policy framework for the assessment and/or reimbursement of technologies on an AA pathway, making clear how the approach differs from that of a traditional HTA or reimbursement pathway Develop and expand pragmatic approaches to risk-sharing, including alternative payment mechanisms with a focus on what is reasonable given data and evidence limitations
**Companies**
Priority actions Ensure meaningful patient engagement early in clinical development; allow patients to submit data and recommend outcome measures that speak to their lived experiences Provide clear, accessible, and interpretable summaries for patients and clinicians of the benefits, risks, and key uncertainties associated with a drug approved under accelerated or conditional regulatory pathways Update regulators and HTA/payer organizations on a regular basis about the status of the clinical development program for a drug being considered under an accelerated or conditional regulatory pathway Reach agreement on the nature and timing of confirmatory evidence well in advance of an anticipated AA regulatory decision Participate in early advice programs and publicly disclose the results of those discussions Longer-term modifications Collaborate with payers in the development of innovative payment models Implement company-specific “horizon scanning” for the HTA and payer community so that priorities, constraints, and requirements can be well understood prior to licensing
**Patients and clinicians**
Priority actions Clinicians have a responsibility to be well informed about the benefits, risks, key uncertainties, and timetable for confirmatory evidence associated with an AA drug, and to ensure that patients understand the impact of all of these elements on the decisions regarding when to pursue treatment, how to monitor their experience, and when to stop taking the drug if necessary Patient organizations should ensure transparency about the sources of their funding, following disclosure rules that are similar to those employed by other players in the AA ecosystem
Longer-term modifications Clinicians should embrace their advocacy role in partnership with interested patients to support early access to medications receiving accelerated or conditional regulatory approval

Abbreviations: AA, accelerated access; HTA, health technology assessment.

## Key takeaways and discussion

The discussions that took place at the 2023 Adelaide Workshop were an important step to an ongoing, complex conversation on how best to coordinate and facilitate AA pathways. Workshop attendees from diverse country and stakeholder perspectives generally agreed that patients with severe or life-threatening conditions should have rapid access to new medicines that could provide a significant benefit, and that early access should not obstruct full assessment of the medicines' safety and efficacy. Bellberry developed a meeting statement (available online and shown here in Table 3) to distill from the discussions some key principles and guide future discussions on this topic.^12^ The meeting statement should not be construed as an endorsement by any individual or organization that participated in the workshop, but as a summary of the key considerations that arose from the discussions.

**Table 3.** Meeting statement.

**Background** Accelerated approval pathways for medicines meeting certain conditions are now offered by a number of regulatory authorities around the world. These pathways have been the subject of much debate in recent years, including how they should be applied, and how patient access (which for most patients depends on reimbursement or coverage decisions) to medicines approved through them can be achieved in a fair and timely manner. Bellberry convened an international meeting of stakeholders in June 2023 to discuss these issues. The meeting gave rise to an energetic and constructive debate, a report of which can be found on the Bellberry website. This statement is Bellberry's attempt to distill and share with others some key principles. While it takes on board views and comments received from meeting attendees, it is not being issued as a formal consensus statement. **Statement** Patients want rapid access to promising new treatments for life-threatening or severe diseases. Health care systems should ensure that there are appropriate mechanisms to respond to this expectation. Many regulators provide accelerated approval pathways, but accelerated regulatory approval does not necessarily lead to accelerated patient access. Accelerated patient access requires cooperation and coordination between the regulator, payers and HTA authorities, those developing and marketing new medicines, and patients and other stakeholders, to achieve an accelerated access pathway. Accelerated access pathways should be designed to provide rapid and equitable access to medicines that are judged reasonably likely to provide significant benefit to patients with life-threatening or severe conditions for which there are currently no effective alternative treatments or there are remaining and significant unmet needs, while supporting the expeditious collection of data for a full assessment of safety and effectiveness. **Developing effective accelerated access pathways is a shared responsibility between:** Regulators who should: apply the accelerated approval pathway only to products that meet agreed criteria for entry establish clear and transparent frameworks for their initial assessments of likely benefits, harms, and uncertainties, and for the collection of further data for re-assessment and confirmation of benefit provide public explanations of their scientific analysis and justifications for their decisions Those developing and marketing new medicines who should: put forward appropriate products for consideration take account of the evidence requirements of payers and HTA bodies when designing clinical development programmes, recognizing that uncertainty about benefits will affect willingness to pay in many jurisdictions provide all relevant data to patients and clinicians, regulators, HTA bodies, and other decision-makers diligently and expeditiously complete confirmatory clinical trials support collection of real-world and other data that can further reduce key remaining uncertainties Patients and clinicians who should: discuss all treatment options carefully, including uncertainties in the potential benefits and risks support the completion of confirmatory clinical trials and collection of additional data to reduce uncertainty be aware that coverage/reimbursement for promising medicines may need to be reviewed if the expected benefits are not confirmed Governments that should: promote and incentivize cooperation and coordination between regulators, payers and HTA bodies, and industry develop policies that promote agreed ethical principles and equity of access for all patients, present and future, to all safe and effective treatments that they need, regardless of clinical condition and means develop policies that promote evidence generation and sharing of information between stakeholders to reduce decision-makers' uncertainty about the benefits, risks and value of medicines, particularly those evaluated via accelerated access pathways **Conclusion** The characteristics of an effective accelerated access pathway will vary between jurisdictions. Key stakeholders (including patients) within a jurisdiction need to work together to build a system that is appropriate to their context. The goal of this statement is to suggest principles for consideration. In most cases, it should be possible to achieve an effective system through cooperation and coordination within the roles and remits currently defined for the regulator, payer(s) and HTA body(ies) in a jurisdiction. While these are local responsibilities, global cooperation has an important role to play in sharing information and learning, and in enhancing efficiency, understanding, and trust.

Abbreviation: HTA, health technology assessment.

Many actionable ideas for the improvement of the AA system were also shared, including ways to enhance coordination between stakeholders. A total of 11 key takeaways were identified from the workshop, as listed below.

- Patients should have rapid access to medicines that are judged reasonably likely to provide benefit to those with life-threatening or severe conditions for which there are currently no effective alternative treatments or there are remaining and significant unmet needs.
- Rapid patient access to a medicine should not obstruct a full assessment of safety and effectiveness of that medicine.
- Concerns about accelerated/conditional approval pathways include the types of medicines considered candidates for such programs, the criteria used to judge “reasonably likely” benefit, the quality of evidence available at the time of approval, and the lack of enforcement of requirements for confirmatory trials.
- The effectiveness of accelerated patient access programs depends on cooperation and coordination between those developing and marketing new medicines, regulatory authorities, and HTA and payer bodies.
- Governments have significant roles to play in the coordination of an effective accelerated access framework, including reducing barriers to communication between stakeholders, providing appropriate incentives for participation in and adherence to accelerated or conditional pathways, and supporting collection of useful data.
- There are concerns that many HTA and payer bodies do not have explicit policies and methods to respond to medicines approved via accelerated or conditional pathways, resulting in unnecessary delays to patient access.
- HTA bodies should find opportunities to collaborate with each other in ways that will make methods and processes more consistent for consideration of medicines approved via accelerated or conditional pathways.
- Companies developing and marketing new medicines would benefit from interactions with HTA and payer bodies as well as regulatory bodies throughout the product lifecycle, and should support efforts to align on outcome measures, collect additional data to reduce uncertainty, and adhere to requirements to complete confirmatory studies in an expeditious manner.
- Patients and clinicians are key partners in the design of trials, determination of technology value, and the collection of further data to assess safety and effectiveness; they should be involved at multiple stages of clinical development and encouraged to support such work.
- Companies developing and marketing new medicines should consider the initial uncertainty associated with an accelerated regulatory approval in pricing policy, and work closely with HTA and payer bodies to facilitate the collection of additional data to reduce uncertainty and explore the potential to share financial risk via novel reimbursement mechanisms.
- An effective AA program needs to reflect the remits and policies of relevant bodies within each jurisdiction in relation health and innovation, equity of access to health care, and underlying ethical principles.

It was clear from these discussions that identification of the issues and areas for improvement, as well as generating a statement of overarching principles and stakeholder responsibilities, was a necessary start. However, with no single party tasked with improving the AA framework, multi-stakeholder efforts will be required to implement meaningful change. Dissemination materials that workshop attendees could take forward, such as a summary slide presentation, “FAQ” documents, and infographics, may help with broader education. Creation of a digital “community of practice” could serve as a forum for individuals from multiple perspectives to interact, share resources and best practices, and develop interest groups, although such a forum will require funding and a commitment from a convening organization.

## Supplementary Material

qxae069_Supplementary_Data
